# Consumption Expenditures in Austria & Germany: New Evidence based on Transactional Data

**DOI:** 10.1515/ger-2024-0110

**Published:** 2025-07-28

**Authors:** Winfried Koeniger, Peter Kreß, Jonas Lehmann

**Affiliations:** Department of Economics, University of St. Gallen, SEW-HSG, Rosenbergstrasse 22, 9000 St. Gallen, Switzerland; CESifo, CFS, IZA, Swiss Finance Institute, Zürich, Switzerland

**Keywords:** consumption expenditures, transactional data, Austria, Germany, C80, D12, E21

## Abstract

We analyze the novel transactional card expenditure data for Germany and Austria provided by Fable Data. We describe key features of the data in terms of the coverage of expenditure items, payment channels, and the distribution of expenditures across regions and time. We highlight strengths and limitations of the data, comparing them to representative but more consolidated lower-frequency information from external data sources. We find very similar expenditure patterns in Germany and Austria. We illustrate the granular, higher-frequency information across expenditure items and locations, analyzing weekday spending patterns and the evolution of consumption expenditures during the COVID-19 crisis and beyond.

## 1 Introduction

Monitoring economic activity and analyzing the effects of economic policy both benefit from high-frequency (daily) data that are quickly available. Transactional data have these desirable features. Because of their superior timeliness and granularity they complement macroeconomic data and surveys from traditional sources, which typically have a monthly or quarterly frequency and are released with a substantial time lag.

We contribute to the literature by describing and validating a novel transactional data set of card transactions for Austria and Germany from Fable Data, and by providing novel stylized facts on the evolution of consumption expenditures during the sample period 2018–2023.1Fable Data provides transactional data also for European countries such as France, Italy, Spain, and the U.K. We first illustrate the variation of the data across time, geographic locations and expenditure items in Austria and Germany. These are two important euro area countries, for which data on expenditures are scarce so that the Fable Data provide a wealth of new information given the regional granularity and daily frequency of the data. We find that the expenditure patterns in the Fable Data are very similar in Austria and Germany. This feature may suit researchers who plan to analyze the effect of a national policy in one of the two countries because observations for the respective other country, or for subregions of it, may provide for a useful comparison (e.g. Koeniger and Kress 2024).

We validate the transactional data for Austria and Germany by comparing the expenditures based on the transactional data to their expenditure counterparts in more consolidated, lower-frequency data sources, which have the purpose to provide representative information on consumer spending. This allows us to illustrate the strengths and limitations of the Fable data set.

We then provide new evidence on the expenditure changes during the last economic crisis, associated with the COVID-19 pandemic, and beyond. Because the analyzed sample period extends to the end of 2023, we can distinguish which changes of expenditures during the pandemic have been persistent and which ones have been transitory.

We find substantial shifts of expenditure across locations and within the consumption basket during the COVID-19 pandemic in Austria and Germany. The shifts across locations have been quite heterogeneous: persistent for some locations, with significant effects three years after the pandemic started, and more temporary for others. The shift of expenditures within the consumption basket has been more short-lived, at least across the expenditure categories we consider in the data. We illustrate that the true size of these shifts is underestimated if expenditure weights are not computed at least at a monthly frequency. Interestingly, the shifts are not associated with changes in the dispersion of total consumption expenditures across cards in either of the two countries during the sample period.

We also illustrate the potential of the expenditure data at a daily frequency, showing the extent to which weekday spending patterns differ across expenditure categories, and how they are affected by discounts in the week of Black Friday. These results highlight the potential of the data for understanding strategic decisions of both firms and households that affect consumer spending.

Our analysis proceeds in the following steps. After a brief review of the related literature, we describe the Fable Data in Section 2. We then compare the data set with other data sources in Section 3, highlighting some strengths and limitations which may be helpful for potential users. In Section 4, we use the data to analyze expenditure shifts in Germany and Austria in the recent economic crisis, triggered by the COVID-19 pandemic, and beyond. In Section 5, we highlight the potential of the granular data at a daily frequency by analyzing weekday spending patterns across expenditure categories, and during the week of Black Friday. We conclude in Section 6.

### 1.1 Related Literature

Our paper relates to the literature which uses high-frequency transactional data to analyze consumption patterns of households, surveyed in Baker and Kueng (2022). Gathergood et al. (2020), for example, use the Fable Data for the U.K. to study consumption responses to pandemic policies, Chetty et al. (2020) use transactional data to analyze the consequences of the pandemic for the U.S., and Cevik (2023) exploits data on debit and credit card transactions to analyze the effect of the pandemic on expenditures in the Baltic countries (see also the references therein for further literature). Buda et al. (2022) and Carvalho et al. (2021) use transaction data from cards and bank accounts to analyze consumption in Spain and its responses to monetary policy shocks (Buda et al. 2023). Cabral et al. (2021) analyze the evolution of consumption expenditures during the pandemic in Portugal. Bounie et al. (2023) use data on bank card transactions in France to document the changes in expenditures and geographic mobility of consumers during the pandemic. Brown et al. (2023) describe transactional data and the associated use cases for Switzerland. Compared to most of the literature on the pandemic, the longer sample period, that extends to the end of 2023, allows us to gauge which of the expenditure changes during the pandemic have been persistent and which have been transitory.

For Austria and Germany, a literature is emerging which uses transactional data to improve forecasts (Forné and Lehmann 2023), to analyze the effect of dividend payments on expenditures (Bräuer et al. 2022), to analyze the effect of the temporary VAT cut in 2020 or the effect of the child bonus on consumption expenditures (Bachmann et al. 2024; Goldfayn-Frank et al. 2022; Koeniger and Kress 2024), to analyze the transmission of monetary policy (Grigoli and Sandri 2022), and to analyze the effect of shifts in the consumption basket on the measurement of inflation (Grigoli and Pugacheva 2024).2
Felbermayr et al. (2021) have used granular regional data to track the spread of the pandemic from Austria to Germany. The analysis of Forné and Lehmann (2023) uses data on transactions in retail and hospitality from Mastercard SpendingPulse™. Bräuer et al. (2022) use transactional data on expenditures from bank accounts in Germany, and Bachmann et al. (2024) or Goldfayn-Frank et al. (2022) combine scanner data with survey data. Grigoli and Sandri (2022), Grigoli and Pugacheva (2024) and Koeniger and Kress (2024) use the Fable Data, which we analyze and validate in detail in this paper. We consider this an important contribution in itself because we expect more researchers to make use of the data in the years to come.

In contemporaneous independent work, Askitas et al. (2024) build on Grigoli and Sandri (2022) and construct an aggregate expenditure index for Germany based on the Fable Data, comparing it to the expenditure time series from the national accounts. This relates to the comparison of aggregate time series which we present in Figure 8 for both Austria and Germany. Our analysis goes much beyond this comparison by validating and analyzing expenditure patterns for different expenditure items and payment channels. We also take advantage of the granular regional information for cardholders and merchants, illustrating the distribution of expenditures across geographical regions over time.

Our results on the evolution of inequality in consumption expenditures in Germany relate to recent work by Bönke et al. (2015), Fuchs-Schündeln et al. (2010), and Hufe et al. (2018). Our analysis of the evolution of consumption expenditures during the pandemic relates to Bernard et al. (2020) who investigate the effect of the pandemic on consumption *plans* in May 2020, using the Bundesbank Online Panel survey for households. We present results on the evolution of consumption expenditures during the pandemic and beyond where the expenditures in our analysis are based on actual transactions. This is an important difference because expenditures during the pandemic have been affected both by changes in the consumption plans as well as by restrictions of supply. It is unlikely that the policies implemented to mitigate the pandemic have been fully reflected in the consumers’ plans in the early phase of the pandemic in May 2020.

For example, Bernard et al. (2020) find that households planned to spend less on vacation in May 2020. We find that households’ actual spending in the category *accommodation* during the pandemic shifted towards the home country but not to the region of residence (see Figure 12 in Section 4.2).

## 2 Data

The data set provided by Fable Data contains transactional data for some European countries. Access to the data is granted by Fable Data on a discretionary basis within their *Data for Good* initiative. Fable Data is a data intermediary which sources the data from financial organizations (e.g. banks, card issuers, open banking fintechs). The data set is growing over time as Fable updates the data set, adding transactional data for more countries or for longer time spans. In this paper, we present the data for Austria and Germany in the period from January 1, 2017 to December 31, 2023.3Before 2017, the sample of cards with the associated transactions in the Fable Data still grows at a very high rate for Austria and Germany. In particular, the size of the sample for Austria more than doubles between 2016 and 2017.


The Fable data set contains issuing data, i.e. data from the card issuer. We thus observe expenditures per card over time, at the point of sale (PoS) or through ecommerce (ECOM), as well as cash withdrawals. We focus on expenditures made with cards issued to households residing in Austria or Germany, whether this expenditure is made at home or abroad. The data do not allow for a consolidation of expenditures across cards at the household level but, on average in 2023, households had only 0.82 credit or delayed debit cards in Austria and 0.84 in Germany.4These type of cards most closely correspond to the cards in the Fable Data for Austria and Germany. See Appendix A.6.3 for further details on the data sources. These are much less cards per household than in the U.S. where the average consumer had 3.9 credit cards in 2023.5Statistic provided by Experian, accessed in April 2025.


Compared with existing survey data on expenditures, the transactional Fable Data are available at higher (daily) frequency and at a finer regional level (postcode). Expenditures based on the available transactions are arguably captured with less measurement error than in data based on surveys but the coverage of expenditures across items is somewhat smaller than in survey data because households pay for certain expenditures with bank transfers or cash.

How do the Fable Data compare to other types of transactional data? Scanner data have the advantage that they contain very detailed information about the purchased products but typically focus on retail expenditure items. Bank account data instead potentially cover all items contained in the consumption basket but only have information on coarser expenditure categories. Furthermore, many transactions may not be categorized at all (35 % in the data set used by Bräuer et al. 2022). Instead, the transaction attributes recorded for each transaction in our card-expenditure data imply that the merchant-category code (MCC) is available for almost all transactions.6Only 9 out of 100,000 transactions do not have information on the MCC for Germany, and 3 out of 100,000 for Austria. The statistics are of the same order of magnitude if we weigh by transaction volume. Information on the specific merchant is less complete, as the merchant associated with a transaction cannot be identified without further imputation for roughly one third of the transactions in the data.

In terms of coverage, the card transaction data provided by Fable Data are somewhat in-between other types of transactional data. The coverage of expenditure items is broader than in typical scanner data. The merchant category code associated with each transaction, however, only allows to categorize expenditures into relatively broader categories. Furthermore, the card expenditure data typically do not contain expenditures for housing rents or other expenditure items, which are paid with direct debit. These items instead are contained in bank account data. Bank account data or fintech data, which pool information from multiple accounts, also have the advantage that income inflows and expenditure outflows can be analyzed jointly, and they may have more demographic information on the households. A common challenge for all of these relatively new transactional data sources is that researchers have to find out how representative the data are for the population.

This is why we validate the Fable Data by comparing it to expenditure data which are collected with the goal to be representative for the population. We elaborate on the pros and cons of the transactional Fable Data in Section 3, where we compare the Fable Data to such external data sources. Baker and Kueng (2022) provide further discussion of the strengths and weaknesses of transactional data.

### 2.1 Data Attributes

The Fable Data provide information on the age and the location of the cardholder, the location and classification of the merchant selling the good or service, and the amount paid. We also merge the average income at the county level from *Statistik Austria* and *Destatis* to the data set, as the income information in the original Fable Data is only available in terms of income bands, albeit at the postcode level.

The data set contains information on expenditures made with a card (the variable *spend-out*) and transfers to the card (the variable *spend-in*), possibly related to refunds.7Transfers to the card may also occur to provide resources for planned future purchases. The *spend-in* amounts are negligible for categories such as groceries, fuel or gastronomy but more relevant for clothing. The percentage of *spend-in* amounts relative to *spend-out* amounts across expenditure categories in the sample period is 5.7 % both in Austria and Germany. Given that the relationship is quite stable over time and *spend-in* amounts cannot be linked in a straightforward manner to the corresponding *spend-out* transaction, we abstract from *spend-in* amounts in the following. For specific episodes and expenditure categories *spend-in* amounts may need to be accounted for.8For example, expenditures for clothing through ecommerce increased during the pandemic, increasing temporarily both *spend-out* and *spend-in* amounts for clothing. Only considering the *spend-out* amount would then imply that the increase of *spend-out* expenditures for clothing would be considerably larger than the expenditure increase net of refunds.


Table 1 displays summary statistics for main variables of interest.9We apply population weights when computing the average of the mean county-level income across counties. We focus on cards that are active at least once in a given year. We provide a more comprehensive list of the variables in the Fable data set in Table 5 in Appendix A.1.

**Table 1:** Summary statistics by country.

Variable	Germany					Austria				
	Mean	Std dev				Mean	Std dev			
Age	45.09	14.68				44.64	14.03			
Income (in 1,000€)^a^	34.69	4.21				29.22	2.93			
# active cards per year (in 1,000)	1,099	209				78	21			
# active cards per inhabitant	0.014	0.005				0.009	0.003			
	**Mean**	**Std dev**	**P10**	**P50**	**P90**	**Mean**	**Std dev**	**P10**	**P50**	**P90**
Expenditure per card (€)^a^	3,010	4,410	150	1,533	7,366	3,022	4,509	122	1,461	7,753
# transactions per card	43.4	70.4	2	19	109	51.6	81.5	3	24	129
# distinct merchants per card	7.35	7.33	1	5	17	7.52	7.02	1	5	17
# distinct MCCs per card	9.72	9.61	1	7	23	11.3	10.5	1	8	26

Notes: ^a^Average annual amount in the sample period 2017–2023. For income, the average is the population-weighted average of the mean income in each county. In the columns with header *P10*, *P50*, and *P90*, we report the value of the variable of interest at the 10th percentile, median, and 90th percentile of the distribution, respectively. Mean expenditure per card refers to the average of the total annual expenditure per card, where we focus on all cards that are active at least once in a given year. Active cards per inhabitant refers to the number of cards that are active at least once per year, relative to the population size at the end of the respective year. Sources: Fable Data, Statistik Austria and Destatis (*Lohn- und Einkommensteuerstatistik*).

The summary statistics in Table 1 are similar for Austria and Germany. The top panel of Table 1 shows that cardholders are on average 45 years old and live in a county in which average income is 30–35 thousand euro. Table 6 in Appendix A.1 shows that the age distribution of the card users in the Fable Data is quite representative of the population but for the group with ages above 70, for which card usage is relatively less common. On average, 1 million cards are active per year in Germany (80,000 in Austria) in the Fable Data, corresponding to roughly 1 active card per 100 inhabitants.

The bottom panel of Table 1 shows that annual expenditure per active card is 3,000 euro, with a large standard deviation showing that the expenditure amounts are substantially larger for a subset of cards.10This is very similar to the average monthly expenditure reported in Grigoli and Sandri (2022), who use the core panel of consumers in the Fable data set for analyzing the transmission of monetary policy in Germany. The annual expenditure at the 90th percentile of expenditure distribution is about 7,500 euro in Austria and Germany and it is 120–150 euro at the 10th percentile. Cardholders on average make 40–50 transactions per year at 7–8 different merchants in about 10 different expenditure categories, i.e. at merchants with different merchant category codes (MCCs).11Note that the transactions in the Fable Data contain a group with *untaggable* merchants. For these transactions, we cannot distinguish the merchants but we can distinguish the MCCs. Again there is substantial variation in the sample. At the 90th percentile of the respective distribution, cards are used for more than 100 transactions at 17 different merchants in more than 20 different expenditure categories. Only a few transactions are made instead at the 10th percentile of the transaction distribution.

The summary statistics show that the Fable Data for Austria and Germany require either aggregation across cards in geographical areas (municipalities, counties or regions) and time intervals (weeks, months or quarters), or a restriction of the sample to a subset of more frequent users to obtain a broad coverage of expenditure items.

### 2.2 Expenditure Items

**Figure 1:**
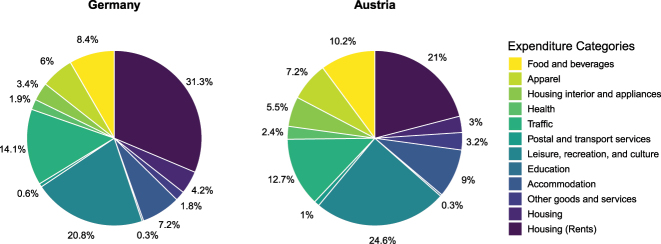
Expenditure shares in Germany and Austria. Notes: Expenditure shares, after accounting for the spending on housing (rents) using official statistics. Sources: Fable Data, Destatis (*Ergebnisse der Laufenden Wirtschaftsrechnungen (LWR) – Haushaltsbuch*), and Statistik Austria (2022): *Volkswirtschaftliche Gesamtrechnungen 1995–2022, Hauptergebnisse*.

Figure 1 displays the expenditure shares in the Fable Data. We use COICOP categories to group the expenditure items so that the shares are more easily comparable with those from official statistics.12Note that when we compare expenditures from Fable Data with consumption time series, we apply a filter to the expenditures in the Fable Data to ensure that these expenditures have a counterpart in the consumption measure of the national accounts. For this purpose, we also add expenditures on housing (rents) from the official statistics, which we do not observe in the Fable Data and which account for 22 % of expenditures in Austria and 33 % in Germany.13In Figure 7 in Section 3 we compare the consumption expenditure shares in the Fable Data with official statistics. The figure shows that, besides housing, the categories leisure, recreation and culture (21 % in Germany and 24 % in Austria), traffic (14 % in Germany and 13 % in Austria), food and beverages (8 % in Germany and 10 % in Austria) and apparel (6 % in Germany and 7 % in Austria) account for about half of the expenditures.

Food and beverages are an example for a category of non-durable consumption goods, apparel contains expenditures for clothing, which is an example for a semi-durable good, and leisure, recreation and culture includes durables such as electronics. We provide a classification of the expenditures in the Fable Data into non-durables, semi-durables and durables in Table 7 in Appendix A.1.

### 2.3 Payment Channels

**Figure 2:**
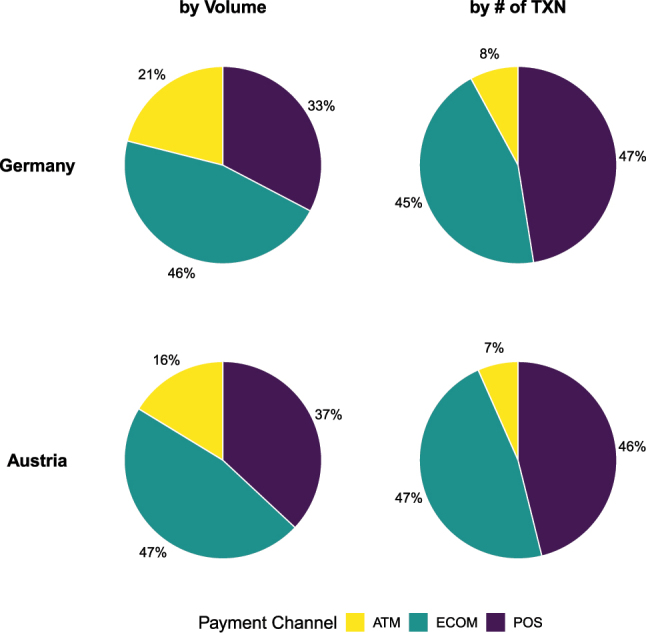
Expenditure shares by payment channel. Notes: Shares by transacted volume and number (#) of transactions. ATM, cash withdrawals; ECOM, ecommerce; POS, point of sale. Source: Fable Data.

Figure 2 shows the shares by payment channel in the sample period. The left panel shows the shares of the transacted volume, and the right panel shows the shares of the number of transactions. The figure shows that the cards in the Fable Data are also used to obtain cash. 7–8 % of the transactions are associated with cash withdrawals, and these withdrawals tend to be larger amounts so that the share increases to 16–21 % if the amount is considered. Indeed, some cards in the Fable Data are used in a way more similar to debit cards rather than credit cards, as discussed further in Section 3 when we compare the transactions with official statistics.

Transactions in ecommerce and at the point of sale (PoS) account for the same share of transactions. The transactions in ecommerce tend to be larger, however. They account for half of the expenditure amounts, which is larger than the share accounted for by PoS expenditures (a third).

### 2.4 Expenditure Time Series

**Figure 3:**
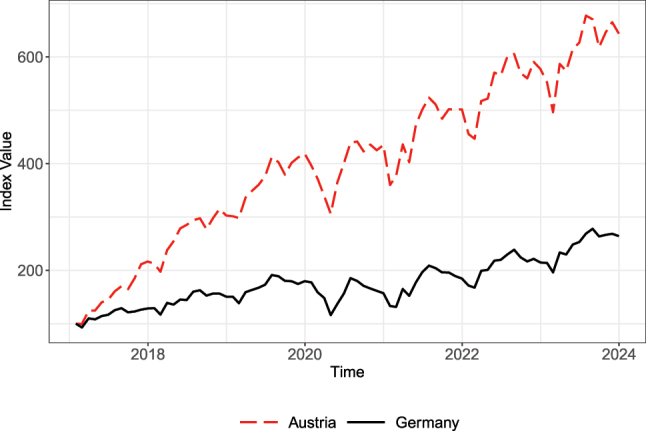
Time series of expenditures in Germany and Austria, not controlling for sample growth. Notes: The time series are normalized to take the value of 100 at the beginning of the sample period. Source: Fable Data.

Figure 3 shows the time series of expenditures including cash withdrawal. The time series are indexed to 100 at the beginning of the sample period. Figure 3 shows a strong upward trend, which is driven by the increase in the number of cards in the sample. The expenditures covered in the Fable Data increase sixfold in the Austrian sample and almost threefold in the German sample.

To account for the sample growth in the analysis, we thus focus on expenditures per active card, where cards are active if they are used at least once per year. We will compare the time series based on the expenditure per active card with time series from other data sources in Section 3.14Depending on the application, the data could be smoothed by constructing one-sided moving averages as in (Buda et al. 2023), or by averaging based on overlapping samples as in Aladangady et al. (2022). The pros and cons of these alternatives depend on the application. If capturing weekly fluctuations in the number of active cards is important for the analysis, for example, smoothing the sample of active users may not be the best alternative.


### 2.5 Regional Expenditure Patterns

To illustrate regional expenditure patterns, we aggregate the transaction-level data to NUTS3 regions. NUTS3 regions correspond to counties or districts (*Landkreise* and *kreisfreie Städte* in Germany, or *Bezirke* and *Statutarstädte* in Austria).

**Figure 4:**
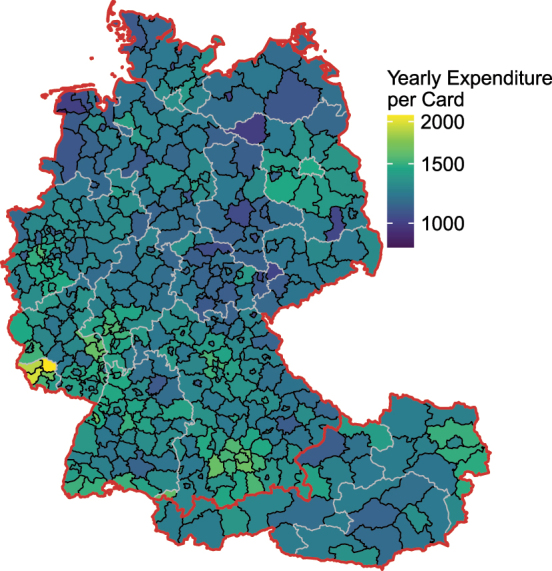
Regional distribution of expenditure across NUTS3 regions. Notes: Average annual expenditures per active card by NUTS3 region in the sample period 2017–2023. Source: Fable Data.

Figure 4 shows the regional distribution of expenditures in Austria and Germany. There is substantial variation in the expenditures across counties. The (population-weighted) standard deviation of annual expenditures per active card across counties is 138 euro.15
Figure 6 provides further detail on the number of transactions and the expenditure amount per transaction. It shows, for example, that the larger average annual expenditure in the *Saarland*, visible in Figure 4, is resulting from a more frequent usage of cards rather than spending larger amounts per transaction. Note that in Figures 4 and 6, a card is considered active if at least one transaction in the sample period has occurred. The annual average is calculated as the total expenditures per year in the sample period divided by the number of active users. The different samples of active card users imply different standard deviations across counties in the two figures.
Figure 16 in Appendix A.1 shows that the expenditure shares of active cards across regions as percent of the total expenditures in the respective country are very similar compared to the statistics on regional expenditure shares at the level of granularity provided by the statistical offices.

**Figure 5:**
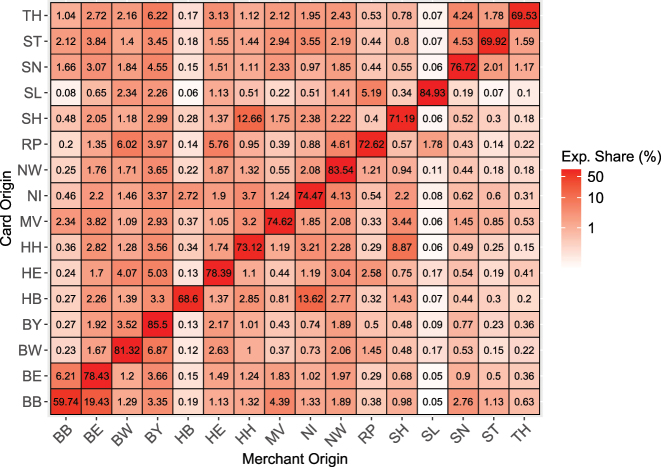
Distribution of point-of-sale expenditures in Germany by merchant location and cardholder residence. Notes: Abbreviations, as used by Destatis, are next to each row and column and denote the sixteen states in Germany. E.g., TH denotes Thuringia, NW denotes North-Rhine Westphalia, BY denotes Bavaria, BE denotes Berlin and BB denotes Brandenburg. Expenditure shares at location of merchants by residence of cardholders, at the state level during the sample period. We report the share of expenditures at domestic merchants so that the percentages in a given row sum to 1 across columns, up to rounding error. Source: Fable Data.

Figure 5 illustrates the distribution of expenditures in Germany by merchant location and cardholder residence, at the state level (*Bundesland*). The corresponding Figure 18 for Austria is provided in the Appendix. These distributions of expenditures across regions may allow to construct disaggregate economic accounts if combined with further disaggregate information (e.g. Andersen et al. 2022).

Cells on the 45-degree line in Figure 5 show the percentage of PoS expenditures of cardholders at merchants in their own state of residence. The figures show that most of the PoS expenditures of cardholders occur in the state of their residence. But significant shares can be spent in other states. For example, the cell in the second column and last row shows that 19 % of the PoS expenditures of cardholders of the state of Brandenburg occur in Berlin during the sample period.

**Figure 6:**
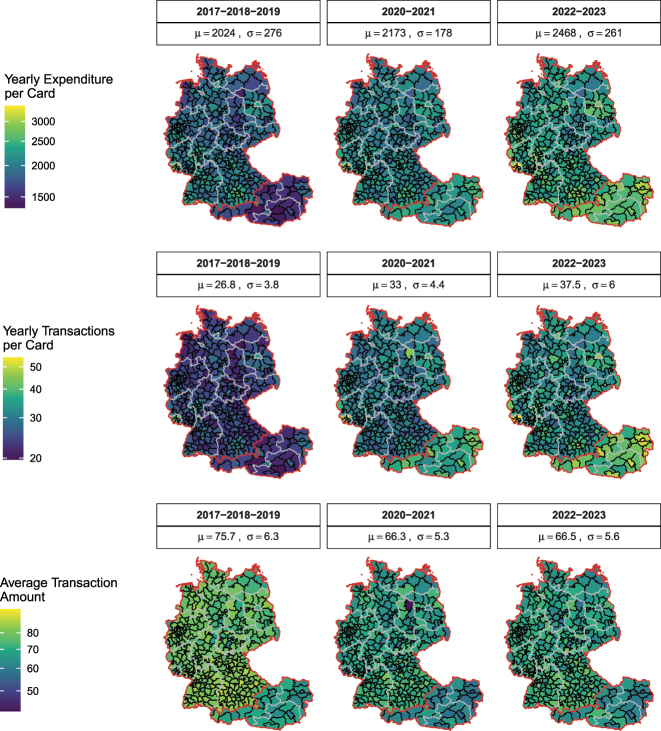
Time variation in regional expenditures in Austria and Germany. Notes: *μ*, mean; *σ*, standard deviation. The top panel shows the average annual expenditures per active card by NUTS3 region for the considered years. The second panel shows the annual number of transactions per card, and the bottom panel shows the average expenditure per transaction. Source: Fable Data.

Figure 6 illustrates that the combination of regional and time variation reveals interesting patterns. The top panel shows the annual expenditures per card, the second panel the annual number of transactions per card, and the bottom panel the average amount per transaction. The three columns split the sample period into the period 2017–2019 before the Covid pandemic, the pandemic period 2020–2021, and the period 2022–2023 after the pandemic.

The figure shows that expenditures increased across all regions because the number of transactions increased as cardholders used the cards more, also for transactions involving smaller amounts. The figure shows that this change has been pronounced in cities during the pandemic, such as Berlin or Vienna.

## 3 Comparison with Other Data Sources

To get a sense of how representative the Fable Data are, we compare them with the income and consumption survey (*Einkommens- und Verbrauchsstichprobe, EVS*), which contains data on expenditures at a lower frequency (every five years) and at a less granular level for regions but has a broader coverage of expenditure items and includes expenditures with cash by expenditure item. We compare the consumption basket in the Fable Data with the basket for Germany based on the EVS, as provided by the Federal statistical office. For Austria, we use the analogous information on the consumption basket provided by Statistics Austria.

We further assess how well the Fable Data captures the time series variation of expenditures for selected expenditure categories, which seem of particular interest and for which data are available from other sources albeit at a lower monthly or quarterly frequency. We consider the expenditure categories *groceries*, *food & beverage services*, *clothing*, *accommodation*, *communication*, *transportation* and *fuel*. The sources of the data used for the comparison are provided in Appendix A.6. For the comparisons, we need to map the MCC codes in the Fable Data to NOGA and COICOP codes. The mappings are available here. The mapping to the COICOP codes also allows to account for inflation in a straightforward way, as inflation is reported by the statistical offices per COICOP category.

Concerning the number of card transactions and expenditures across payment channels, we use statistics reported by the respective central banks to benchmark the Fable Data. Again, the data sources are reported in Appendix A.6.

### 3.1 The Consumption Basket

**Figure 7:**
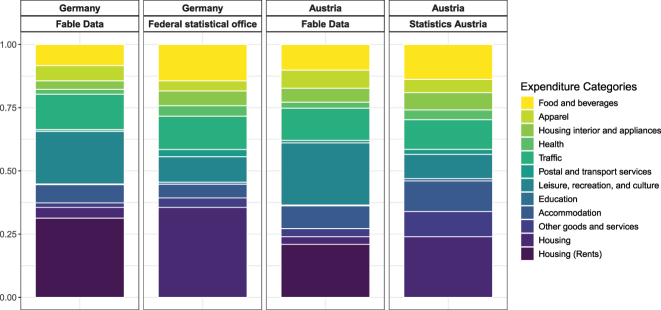
Comparison of expenditure categories (COICOP). Notes: Share of expenditures grouped by COICOP categories. Sources: Fable Data, Destatis (*Ergebnisse der Laufenden Wirtschaftsrechnungen (LWR) – Haushaltsbuch*), and Statistik Austria (2022): *Volkswirtschaftliche Gesamtrechnungen 1995–2022, Hauptergebnisse*.

Figure 7 provides a comparison of the transactional data in the sample period with available official statistics until 2022. For a sensible comparison, we account for expenditures for housing such as rental payments which households do not pay by card and which are added from the official statistics at the bottom of the respective column in the figures. Thus, the two bottom categories for the transactional data (*housing* together with the residual stand-in category *housing(rents)*) add up to the bottom category in the official statistics.16The shares of the other expenditure categories in the transactional data are rescaled to be comparable with the shares reported in the official statistics which contain expenditures on rents. Further information on the data sources of the expenditure statistics in the national accounts is provided in Appendix A.6.1.


Figure 7 shows that the expenditure shares based on the transactional data and the official statistics are highly positively correlated.17Excluding housing, which we have added from the official statistics, the correlation coefficient is 0.74 for Germany and 0.6 for Austria. The share of expenditures associated with leisure and culture or with apparel are somewhat larger in the transactional data whereas the expenditure share of food and beverages is a bit larger in the official statistics.

Overall, the expenditure shares based on the card data are quite comparable to those from the official statistics. In terms of the absolute amount of expenditure in the Fable sample, expenditures made with cards in the sample period 2017–2023, excluding housing, account for 8 % in Germany and 5 % in Austria of the corresponding expenditures reported by the respective statistical offices.18We compare the cumulated expenditures in the Fable Data with the corresponding aggregates in the national accounts. Further details on the national account tables are in Appendix A.6. The Fable Data thus contain a sizable fraction of aggregate expenditures and capture trends and fluctuations that are observable in the aggregate data, as we will see next.

### 3.2 The Time-Series Variation of Expenditures

**Figure 8:**
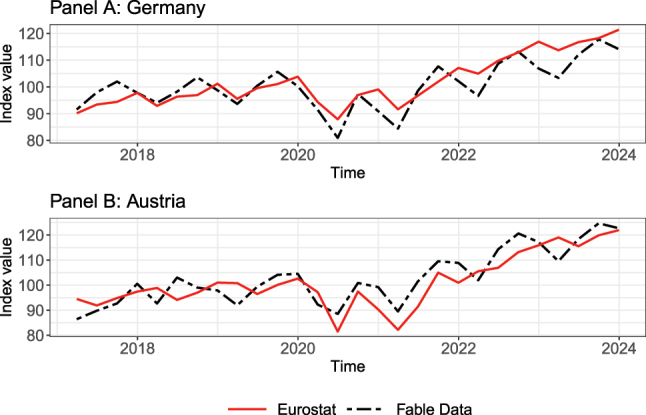
Time series of expenditures in Germany and Austria. Sources: Fable Data and Eurostat time series of final consumption expenditure of households (namq-10-fcs) in euro. The expenditures based on Fable Data are quarterly expenditures in euro per active card. Both time series are indexed to 100 for the average in 2019.

Figure 8 shows that the times series of the expenditures in the Fable data set exhibit very similar trends and fluctuations in Austria and Germany as consumption expenditures in the national accounts. This is remarkable because certain expenditure categories such as housing and energy are not well covered in the Fable data set. Because expenditures in these categories are rather stable over time, fluctuations of expenditures in the Fable Data tend to be more pronounced than fluctuations of consumption expenditures in the national accounts. The correlation between both time series is 0.84 for Germany and 0.86 for Austria. The stronger upward trend after the pandemic relative to the time period until 2020, which is visible in the time series in Figure 8, also reflects the higher post-pandemic inflation.

For a subset of expenditure categories, we compare the time series of expenditures in the Fable Data with revenue statistics from firms that are available at a monthly or quarterly frequency.19
Figure 17 in Appendix A.2 shows the expenditure shares for the subset of the MCC in the Fable Data, for which we provide time series comparisons. These categories account for around 40 % of the expenditures in the Fable Data. Because the revenue statistics contain expenditures by domestic cardholders and foreigners, they may feature different seasonal patterns than the series for domestic cardholders constructed with the Fable Data.20The Fable Data contain issuing data on cards and not acquiring data from merchants. Thus, the data do not allow to track *all* card transactions at a given merchant although the Fable Data contain information on some foreign cardholders. This has to be kept in mind, when comparing the time series. To improve comparability with the revenue statistics, we focus on the domestic expenditures of cardholders in the Fable Data.

**Figure 9:**
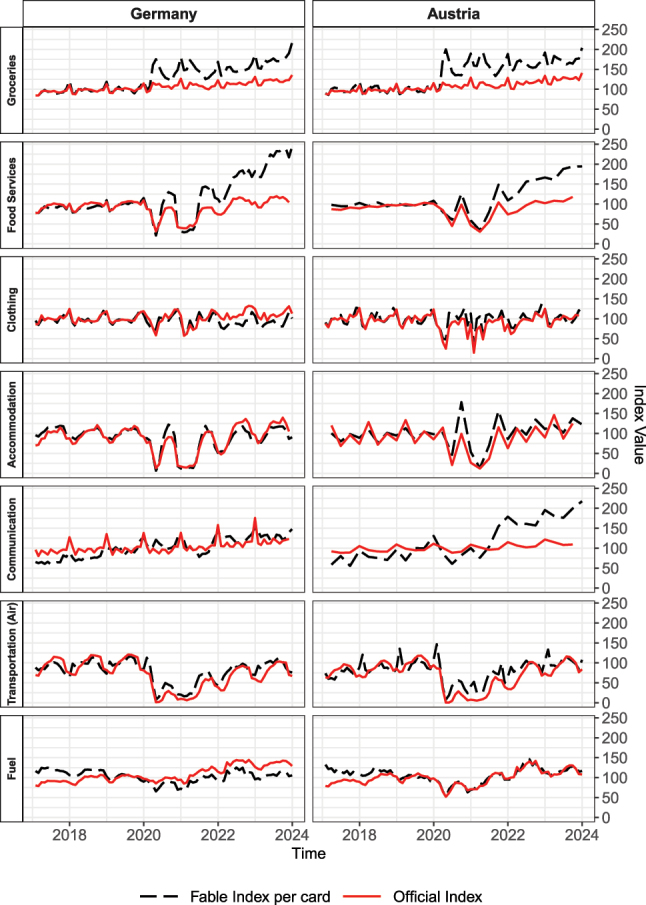
Comparison of time series for selected expenditure categories. Sources: Fable Data; for other data sources see Appendix A.6.

Table 2 displays the correlations of the time variation in the Fable Data and the data from other sources for each of the considered expenditure categories. The main take away is that the time series fluctuations of firm revenues in the reported categories are quite comparable to those of expenditures in the Fable Data. There are some differences, which become more transparent in Figure 9 and are worth discussing because they also illustrate important features of the Fable Data.

**Table 2:** Time series correlations by expenditure category: Fable Data versus other data.

Expenditure category	Germany	Austria
Groceries	0.93	0.87
Food & beverage services	0.74	0.77
Clothing	0.83	0.79
Accommodation	0.92	0.80
Communication	0.66	0.78
Transportation	0.90	0.78
Fuel^a^	0.29	0.78

Notes: ^a^The correlation changes to 0.51 for Germany and 0.76 for Austria if we condition on cards being actively used for fuel expenditure rather than for any expenditure across categories. See the text for further discussion. Sources: Fable Data; for other data sources see Appendix A.6.

For groceries, Figure 9 shows an upward level shift of expenditures made by card during the pandemic that has been persistent over time and is associated with the shift from cash payments to card payments triggered by the pandemic (e.g. Kotkowski and Polasik 2021; Wisniewski et al. 2024).21See Section 4.3 for further suggestive aggregate evidence based on the Fable Data. For food and beverages consumed in restaurants and bars (food services), the shift has taken place a bit later which is associated with supply-side constraints in this expenditure category during the pandemic. The correlation of the time variation in the Fable Data with the revenue statistics has remained high after the shift. Researchers should be aware of these shifts in the Fable Data, especially if they focus on expenditure in these categories.

The time series of the Fable Data and the revenue statistics are very similar and highly correlated for the categories clothing, accommodation, and transport. Some of the differences visible in Figure 9 are associated with expenditures by foreign cardholders, especially in Austria which is a popular destination for foreign tourists particularly in the winter season. These expenditures show in the revenue statistics but not in the expenditures of domestic cardholders in the Fable Data. Depending on the research question, researchers have to be aware of this data feature.

A further important data feature for expenditures on transportation is that the time series from external sources is highly correlated with the Fable Data if we consider air transportation statistics in terms of number of passengers (as done in Table 2 and Figure 9). Expenditures for transport in the revenue statistics also include expenditures by firms for transporting and storing cargo which have quite different dynamics and are not covered well in the Fable Data.

The time series correlation for expenditures on communication is high but Figure 9 reveals some difference towards the end of the sample period in Austria. These differences are associated with how well the MCC in the Fable Data match with the goods and services in communication covered in the revenue statistics. Given that the revenue statistics also cover activities related to the production of communication services and the services contained in this category change over time, finding time series that closely match expenditures in this category in the Fable Data is a challenge.22Using subcategories of the revenue statistics on information and communication that focus on telecommunication or retail sales of information and communication equipment did not further improve the correlation with the variation in the Fable Data reported in Table 2. Furthermore, the Fable Data do not contain expenditures made by direct debit which seem important for this expenditure category.

Finally, expenditures on fuel in the Fable Data are highly correlated with revenue statistics in Austria but less so in Germany. Hence, the fluctuations of expenditures of domestic cardholders in Germany are less strongly associated with the fluctuations of revenues at gas stations than in Austria. The lower correlation for Germany seems to be associated with cards in Germany being used more for expenditures other than fuel over time. Recall that in our benchmark time series, we construct expenditures per active card, where a card can be active in any of the expenditure categories including fuel. Thus, a broader usage of cards across categories may imply that an alternative normalization by active cards, conditioning active card usage on expenditures occurring in the specific expenditure category of fuel, results in a time series that is more highly correlated to the revenue statistics for fuel. Indeed, using the alternative normalization improves the correlation with the revenue statistics from 0.29 to 0.51 for Germany. For Austria instead, the alternative normalization results in a correlation of 0.76 which is very similar to the correlation of 0.78 reported in Table 2. This example illustrates that shifts of card expenditures across expenditure items over time may be important to account for, in certain expenditure categories and countries.

### 3.3 Card Transactions

Table 3 compares the average amount of a transaction and of cash withdrawals made with cards in the Fable data set during the sample period, with the respective amounts for debit or credit cards reported by the Bundesbank and OeNB.

**Table 3:** Transactions and cash-withdrawals: Fable Data versus other data.

	Transaction amount		Cash withdrawals	
	Germany	Austria	Germany	Austria
Fable data^a^	61.73	54.33	191.20	148.97
Debit card	48.30^b^	37.76^c^	235.76^d^	266.30^e^
Credit card	58.23^b^	77.86^c^	167.38^d^	–
Credit card (broad)	69.92^b^	76.32^c^	222.63^d^	197.95^f^

Notes: Averages for active cards in the Fable Data during the sample period. *Credit card* refers to credit cards only whereas *Credit card (broad)* refers to delayed debit cards and credit cards. Statistics for debit and credit cards are from the Bundesbank and OeNB and available for the years indicated as follows: ^a^2017–2023, ^b^2017–2021, ^c^2017–2023, ^d^2022–2023, ^e^2017–2023: *On-Us transactions*, ^f^2017–2023. Sources: Fable Data; Bundesbank, OeNB further documented in Appendix A.6.

Table 3 shows that the amounts transacted with the cards in the Fable data set are similar to those reported by the central banks. As confirmed in conversations with Fable Data, cash withdrawals do not trigger the fees common for typical credit cards for some of the cards in the Fable data set. Thus, it is not surprising that the withdrawn amounts as well as the transaction amounts for the cards in the Fable data set are in between those reported for credit and debit cards reported by the central banks. Table 8 in Appendix A.3 shows that the transaction amounts and the size of cash withdrawals for the cards in the Fable Data have remained rather stable over time, suggesting that the composition of cards has not changed much during the sample period.

### 3.4 Accounting for Structural Changes during the Sample Period

Let us now take stock which structural changes researchers have to account for when working with the Fable Data for Austria and Germany to avoid biased results in the analysis. Shifts from cash to card payments during the pandemic episode, visible in Figure 9 in the two time series of expenditures for groceries and food services, would imply a spurious upward jump in expenditures unless they are accounted for. Cash expenditures are not observed in the Fable Data but for the cash withdrawals, which cannot be linked to specific expenditure categories. Information based on payment diaries provided by central banks may help researchers to correct for such shifts by expenditure category to some extent, albeit at a low frequency. The Bundesbank, for example, provides information on payment behavior and its changes at roughly a two-year frequency (Deutsche Bundesbank 2023).

Furthermore, there may be shifts in the type of cards covered in the sample provided by Fable Data for Austria and Germany. As discussed in Section 3.3, some credit cards in the data set have characteristics similar to debit cards, showing in the transacted amounts and the size of the cash withdrawals. Table 8 in Appendix A.3 shows that these amounts have remained rather stable over time, suggesting that the composition of cards with different features in the Fable Data for Austria and Germany has not changed much during the sample period. Researchers will have to monitor possible changes in the card composition as further data becomes available in future years.

Another issue is sample growth because of the increase in the number of cards, especially at the beginning of the sample period. This would imply a spurious trend in expenditures unless it is accounted for, e.g. by constructing expenditures per active card as discussed in Section 2. As the analysis of the time series for fuel expenditures in Germany has revealed in Section 3.2, accounting for possible shifts in the patterns of card activity across expenditure items can be important for some expenditure categories in some countries.

## 4 Consumption Expenditures during Times of Crisis

We proceed by analyzing shifts of expenditures within the consumption basket and across locations, as well as the evolution of the distribution of the expenditures, during the crisis of the COVID-19 pandemic and beyond. The analysis builds on the results presented in the previous sections for the cross-section of transactions during the sample period. Besides being interesting in its own right, the analysis highlights the value added of transactional data at a daily frequency for researchers and policy makers. We further extend this analysis in Section 5 which zooms in on weekday spending patterns.

### 4.1 Shifts in the Consumption Basket

**Figure 10:**
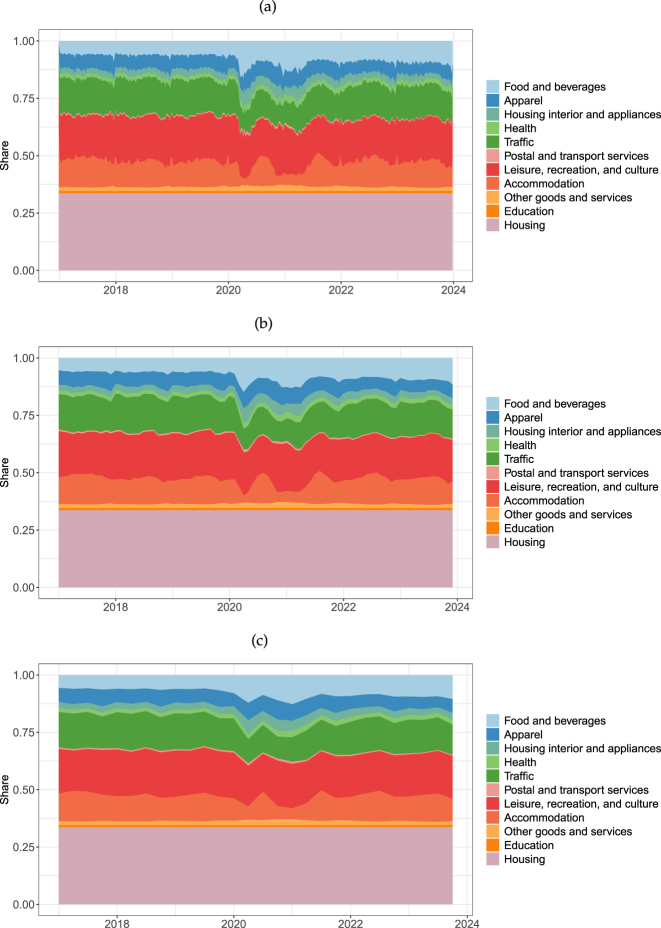
Change of consumption basket in Germany over time. (a) Weekly frequency. (b) Monthly frequency. (c) Quarterly frequency. Notes: Expenditures for the COICOP categories housing and education, which are not covered by Fable Data, do not vary over time in the figure because they are added from the survey *Einkommens- und Verbrauchsstichprobe (EVS)* in 2018 for completeness. Source: Fable Data, EVS.

Figure 10 illustrates seasonal shifts in the consumption basket, such as the surge in expenditure on *apparel* and *food & beverages* during the holiday season. The figure further illustrates the structural shifts during the pandemic, when expenditures in the categories *transport services* almost vanished whereas expenditure on *food & beverages* surged. Figure 19 in Appendix A.4 shows analogous patterns for Austria.

Figure 10 illustrates the value added of expenditure data at a weekly or monthly frequency because the size of the shifts in the consumption basket is partially smoothed out when aggregating expenditures to a quarterly frequency. For example, the standard deviation of the share of expenditure categories such as accommodation or leisure increases by 20 % or 30 % if the shares are measured at a weekly rather than quarterly frequency. Traditional data sources, which do not contain the higher frequency information, compromise accurate measurement which is not only relevant for statistical agencies and central banks, e.g. concerning the weights used for computing inflation, but also for businesses and households concerning optimal inventory management (e.g. Baker et al. 2021).

Because it is very costly to capture the illustrated higher-frequency changes of the consumption basket with surveys, transactional data such as the Fable Data thus can serve as a useful complement to more traditional data sources. Grigoli and Pugacheva (2024) indeed construct inflation time series for the U.K. and Germany taking shifts of the consumption basket into account.

### 4.2 Shifts in the Geographical Distribution of Expenditures

**Figure 11:**
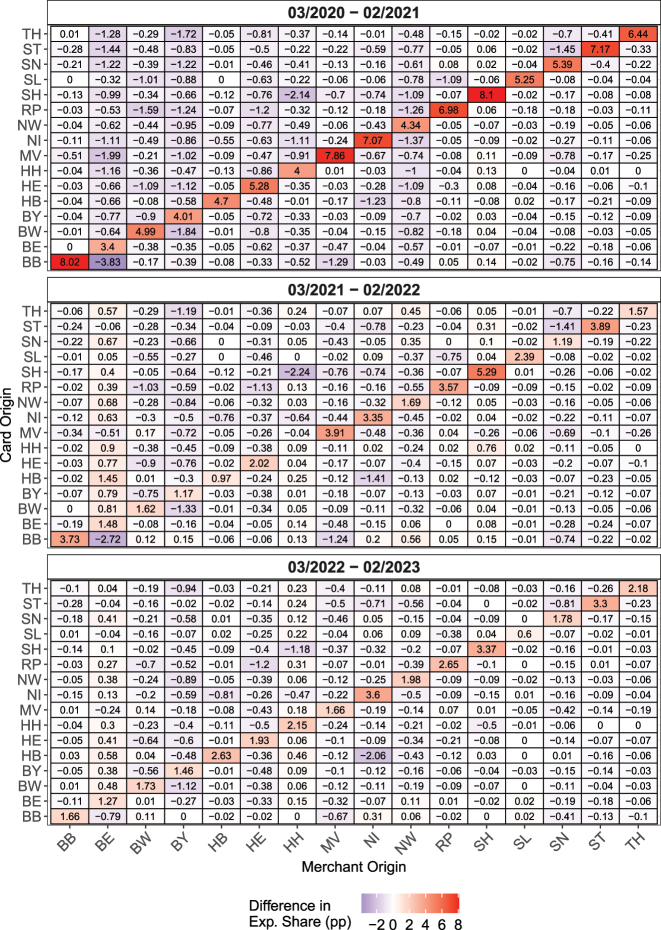
Changes of expenditures by merchant location and cardholder residence, during the COVID-19 pandemic and beyond (Germany). Notes: Changes of expenditure shares in percentage points, at location of merchants by residence of cardholders. The benchmark pre-pandemic period is 03/2019–02/2020. See also the notes to Figure 5. Source: Fable Data.

Figure 11 shows the change of the regional distribution of expenditures during the three years after the COVID-19 pandemic started in Germany, relative to the distribution in the period 03/2019–02/2020, i.e. prior to the pandemic.23See Figure 5 in Section 2.5 for the regional distribution for the whole sample period. The analogous Figure 20 for Austria is in Appendix A.4.

The first, top panel of Figure 11 displays the change of PoS expenditures between the period March 1, 2019 to February 29, 2020 and the subsequent year March 1, 2020 to February 2021. Quite intuitively, PoS expenditures became more local during the pandemic when cardholders spend more in their region of residence, as illustrated by the positive percentage point increases of up to 8 percentage points shown in the cells on the 45-degree line. The negative percentage point decreases in the cells off the diagonal indicate instead a decrease of the share of expenditures by cardholders outside their region of residence. These shifts of expenditure are sizable and show the repercussions of the pandemic for local economic activity. All economically significant changes of 0.05 percentage points or larger are also statistically significant at conventional levels.

These findings are qualitatively similar to Bounie et al. (2023) who show that the mobility of consumers decreased significantly in France during the first year of the pandemic in 2020. Our longer sample period allows us to gauge to which extent these changes have been persistent.

In particular, the second and third panel in Figure 11 provide evidence that expenditures remained more local until 2023 in some locations and hence well beyond the end of the pandemic. Overall, the expenditure shifts across locations have been heterogeneous: persistent for some locations, with significant effects three years after the pandemic started, and more temporary for others. For example, expenditures became more local in both Berlin (BE) and Bavaria (BY) during the first year of the pandemic (see the first top panel of Figure 11). Expenditures of cardholders who resided in the respective other states declined both in Berlin and Bavaria. Whereas these expenditures reverted back their pre-pandemic level in Berlin to a large extent, this was less the case in Bavaria (see the second and third panel of Figure 11). Figure 20 in Appendix A.4 shows that these patterns have been qualitatively similar in Austria, with expenditure shifts in Vienna showing similar dynamics as in Berlin and expenditure shifts in Salzburg showing similar dynamics as in Bavaria.

**Figure 12:**
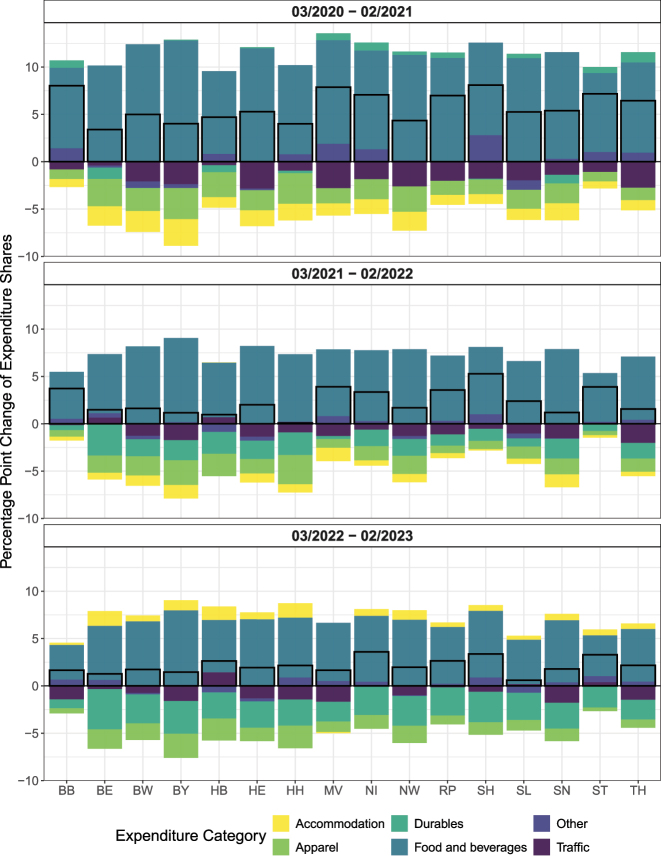
Decomposing the change of expenditure shares of cardholders in their region of residence (Germany). Notes: The black rectangles in the figure illustrate the total change of expenditures. Changes of expenditure shares of cardholders in percentage points, at merchants in the region of the residence of cardholders. The classification of durable expenditure is documented in Table 7, Appendix A.1. Source: Fable Data.

Figure 12 illustrates how different expenditure categories have contributed to the changes of the local expenditures in the region of the cardholders’ residence, i.e. to the changes reported in the cells on the diagonal of the matrix displayed in Figure 11. The black rectangles in the figure illustrate the total change of expenditures. Figure 12 reveals that more local purchases of non-durables such as food (capturing mostly spending in supermarkets) and durables have contributed most to the increase of the home bias of expenditures at the point of sale during the first year of the COVID-19 pandemic. The opposite holds for expenditures on accommodation, traffic, and semi-durables (contained in the category apparel). In the following years, the contribution of some categories, such as durables and accommodation to local expenditures in the region of the cardholders’ residence, changed as the pandemic subsided. Figure 21 in Appendix A.4 shows similar patterns for Austria.

One interpretation for why accommodation temporarily contributed to the spatial dispersion of expenditures during the pandemic is that households substituted away from holidays abroad (which do not appear in the heat maps for domestic expenditures) towards domestic accommodation but not in the cardholders’ state of residence. These results provide an example for how the Fable Data may provide new insights by tracing out the effects of economic shocks and policies over time and across locations.

### 4.3 Shifts in Expenditures by Payment Channel

**Figure 13:**
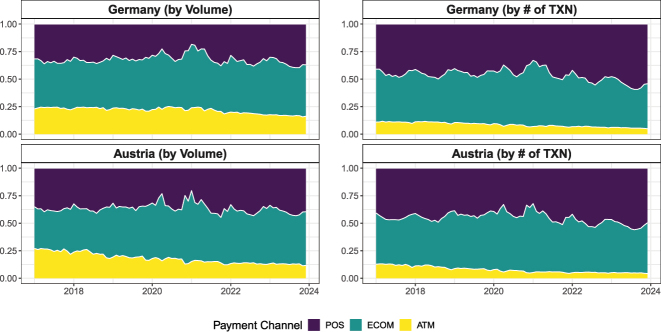
Payment channels. Notes: Shares by transacted volume and the number (#) of transactions at a monthly frequency. ATM, cash withdrawals; ECOM, ecommerce; POS, point of sale. Source: Fable Data.

Figure 13 illustrates at a monthly frequency how transactions across payment channels by domestic cardholders have changed during the sample period. The figure shows the declining share of cash withdrawals and the fluctuations of the share of card transactions in ecommerce and at the point of sale during the pandemic. These trends and fluctuations have to be kept in mind when interpreting the card expenditures over time, in particular for certain expenditure items as discussed in Section 3.2.

### 4.4 Distributional Implications

The Fable Data allow to gauge the distributional implications of the economic crisis associated with the pandemic further. To reduce the effect of cards with little usage and shifts of expenditures into cashless payments on the distributional measures, we construct overlapping samples as in Aladangady et al. (2022) where we focus on cards that are used for at least 50 transactions and four different MCCs and spend at least 1,500 euro on groceries in 13 subsequent months. This sample of more intensive card users accounts for about 3 % of active card users in Austria and Germany, resulting in a sample size of cards well above 10,000 for Austria and Germany in each of the overlapping samples.

The top panel of Table 4 shows that the dispersion of card expenditures is similar in Germany and in Austria during the sample period, in particular for total expenditures.24Note that Meyer and Sullivan (2023) use a subset of consumption items, which are better measured, to compute consumption inequality. See also Hufe et al. (2018), p. 193. Of these items (food at home, rent plus utilities, gasoline and motor oil, the rental value of owner-occupied housing, and the rental value of owned vehicles), however, the Fable Data only contains expenditures on food consumption and gasoline. Given that measurement of expenditures is less of an issue with transactional data, we report dispersion of expenditures for food and for the total expenditures covering those items observed in the Fable Data. The dispersion of food expenditures is slightly higher in Austria compared to Germany, where the difference is more pronounced at the bottom of the distribution as shown by the ratio of the expenditures at the 50th and 25th percentile (p50/p25). The bottom panel of Table 4 shows that the dispersion in terms of the Gini has remained rather stable over time during the sample period.

**Table 4:** Dispersion of expenditures.

	Total expenditures		Food expenditures	
	Germany	Austria	Germany	Austria
Gini	0.47	0.46	0.47	0.49
p90/p25	5.31	5.26	5.60	7.09
p90/p50	2.89	2.75	2.54	2.65
p50/p25	1.84	1.94	2.20	2.67
**Gini**	**Germany**	**Austria**	**Germany**	**Austria**
2018–2019	0.46	0.45	0.47	0.49
2020–2021	0.47	0.45	0.46	0.48
2022–2023	0.48	0.45	0.47	0.47

Notes: Average dispersion of expenditures in the sample period for active cards with at least 50 transactions per year in at least four different MCC and spending of at least 1,500 euro on groceries. p90/p50 denotes the ratio of expenditures at the 90th percentile and the median. The other percentile ratios are defined analogously. Source: Fable Data.

Quantitatively, the dispersion of total card expenditures is in the range of the Gini of 0.41 for total consumption expenditures reported in Hufe et al. (2018), based on the German consumption survey *EVS* in 2013. This is remarkable because the *EVS* covers a broader range of expenditure items including housing, and it covers consumption expenditures based on card and cash payments. In comparison to the *EVS*, the Fable Data also do not allow for a consolidation of expenditures across intensively-used cards at the household level and to account for differences in household size.

Table 4 further shows that the dispersion of food expenditures and total expenditures is quantitatively very similar in the considered sample of intensive card users in the Fable Data, whereas Hufe et al. (2018) document a higher dispersion of total expenditures in the *EVS* in 2013 than of expenditures that are more precisely measured in the *EVS* and include food expenditures. Table 9 in Appendix A.5 reveals that the intensive card-user sample, used for computing the expenditure dispersion in the Fable Data, does not differ much in terms of observable characteristics from the full sample of active cards (used at least once a year), for which we presented summary statistics in Table 1. The intensive-user sample has slightly more card users in cities but is very similar in terms of age and income. This suggests that the similar dispersion of total expenditures and food expenditures reported above does not result from sample selection in these dimensions.

More generally, one has to be careful with interpreting the dispersion of expenditures. Expenditures for food (a non-durable good which fully depreciates over a short time horizon) correspond closely to consumption. This is not the case for durable goods which, by definition, depreciate more slowly. Thus, they generate consumption flows over an extended period of time but expenditures for these durables occur at a specific point in time. The dispersion of total expenditures including durables thus should not be compared to measures of consumption inequality, for which durable expenditures have been converted into durable consumption flows. We leave the further analysis of the expenditure dispersion in the Fable Data to future research.

## 5 Weekday Spending Patterns

We illustrate the potential of the Fable Data for understanding the behavior of households and firms at the daily frequency. We illustrate weekday spending patterns across expenditure categories, and for the week containing Black Friday.

**Figure 14:**
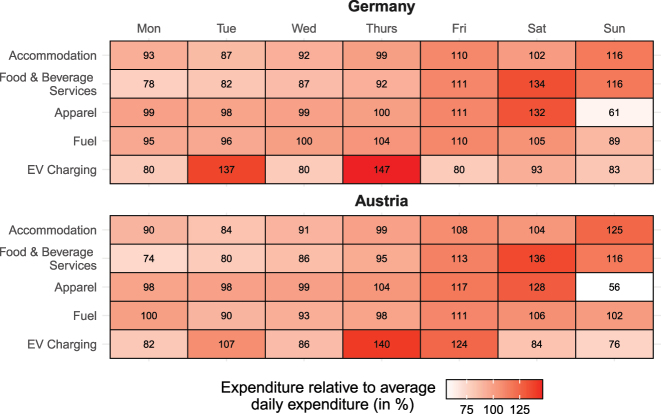
Weekday spending behavior for selected categories. Notes: Expenditures on a respective weekday relative to the average daily expenditure. EV, electric vehicle. Source: Fable Data.

Figure 14 shows that the incidence of expenditures across weekdays differs widely across expenditure categories, both in Germany (top panel) and Austria (bottom panel). As an illustration, the first two rows of each panel show that expenditures for *accommodation* and *food & beverage services* are highest on weekends. The expenditures for *accommodation* are largest on Sunday, on which the expenditures are 16 % (Germany) and 25 % (Austria) higher than the average daily expenditure. The second row shows that expenditures for *food & beverage services* on Saturday are 34–36 % higher than the daily average. Monday and Tuesday are the days with least expenditures for both *accommodation* and *food & beverage services*.

For the expenditures on *apparel* containing clothing, displayed in the respective third row of the top and bottom panel, expenditures are highest on Saturday, with expenditures exceeding the average daily expenditure by 32 % (Germany) or 28 % (Austria). Although apparel can be purchased also through ecommerce, Figure 14 shows that Sunday is by far the day of the week with the lowest expenditures for apparel in Germany and Austria where shops are typically closed on Sundays. The last two rows show expenditures at gas stations and for the recharging of electric vehicles (not at the own residence). Whereas expenditures at gas stations are highest on Friday and Saturday, expenditures for charging electric vehicles are much more concentrated during the week with a peak on Thursday. Owners of electric vehicles can recharge their vehicles at home where they spend most of their weekends on average. Although the transaction volume for the charging of electric vehicles (not at the own residence) in the Fable Data only amounts to 0.2 % of the expenditures at gas stations in both Germany and Austria, the very different weekday expenditure patterns for the charging of electric vehicles suggest that substantial adjustments for gas stations will be required to accommodate these different patterns and accompany the increase in the market share of electric vehicles.

These stylized facts are relevant for understanding decisions of both firms and households. The Fable Data allow to track these patterns over time. As a further illustration, we show the weekday spending patterns around Black Friday, a day on which many retailers offer substantial discounts to start off shopping in the run-up to the holiday season.

**Figure 15:**
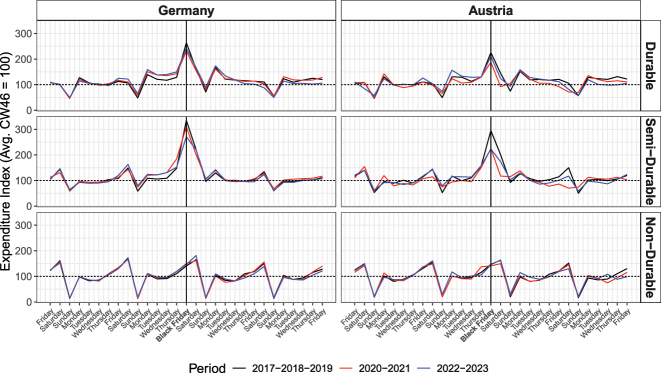
Spending behavior around Black Friday. Notes: Expenditures for durables, semi-durables and non-durables, as defined in Table 7 of Appendix A.1. Expenditures are normalized by the average expenditure in calendar week 46 (in mid November) of the respective year. Source: Fable Data.

**Figure 16:**
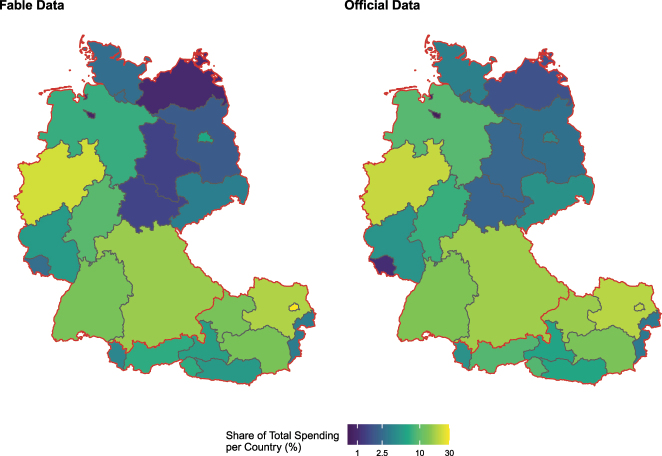
Comparison of the expenditure shares across regions. Notes: The expenditure shares of regions in percent of the respective total in Austria or Germany. Sources: Fable Data and the federal statistical offices (see the description in the text for additional information).

**Figure 17:**
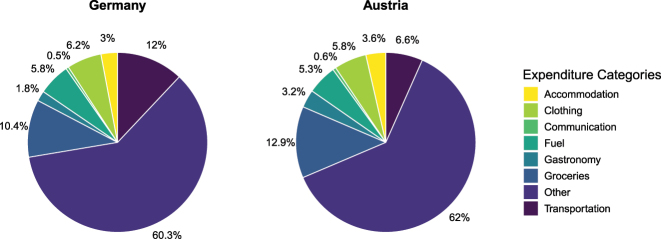
Expenditure shares in the Fable Data based on MCC. Notes: Shares according to the merchant category classification (MCC) for those categories, for which we provide a time series comparison with official statistics on the revenues of firms, which are available at monthly or quarterly frequency. The category *Other* is the residual category containing all other transactions. Source: Fable Data.

**Figure 18:**
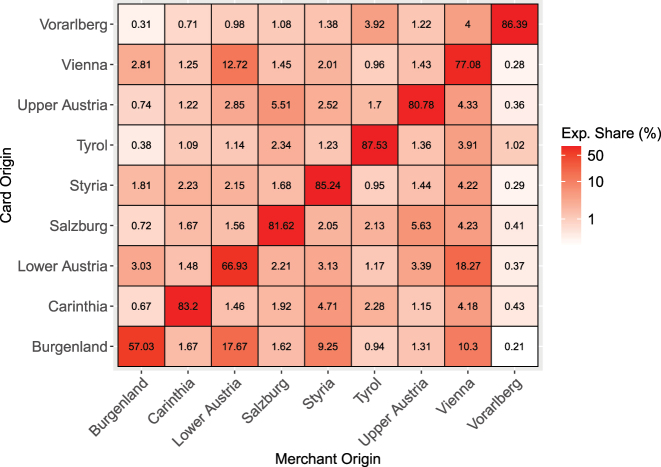
Distribution of point-of-sale expenditures in Austria by merchant location and cardholder residence. Notes: Expenditure shares at location of merchants by residence of cardholders, at the state level during the sample period. We report the share of expenditures at domestic merchants so that the percentages in a given row sum to 1 across columns, up to rounding error. Source: Fable Data.

**Figure 19:**
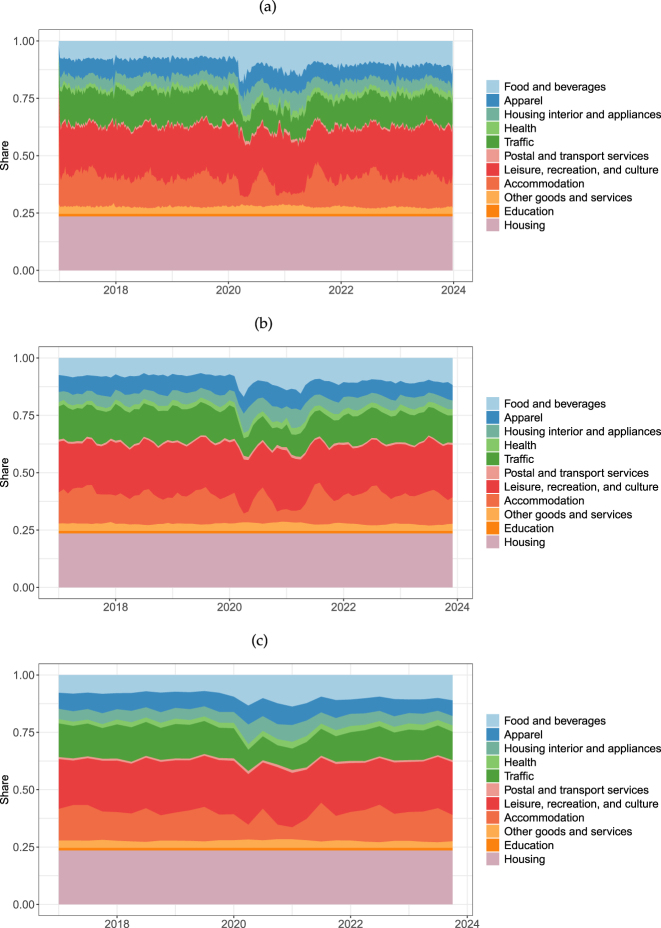
Change of consumption basket in Austria over time. (a) Weekly frequency. (b) Monthly frequency. (c) Quarterly frequency. Notes: Expenditures for the COICOP categories housing and education, which are not covered by Fable Data, do not vary over time in the figure because they are added from the survey *Konsumerhebung* 2019 for completeness. Source: Fable Data, *Konsumerhebung*.

**Figure 20:**
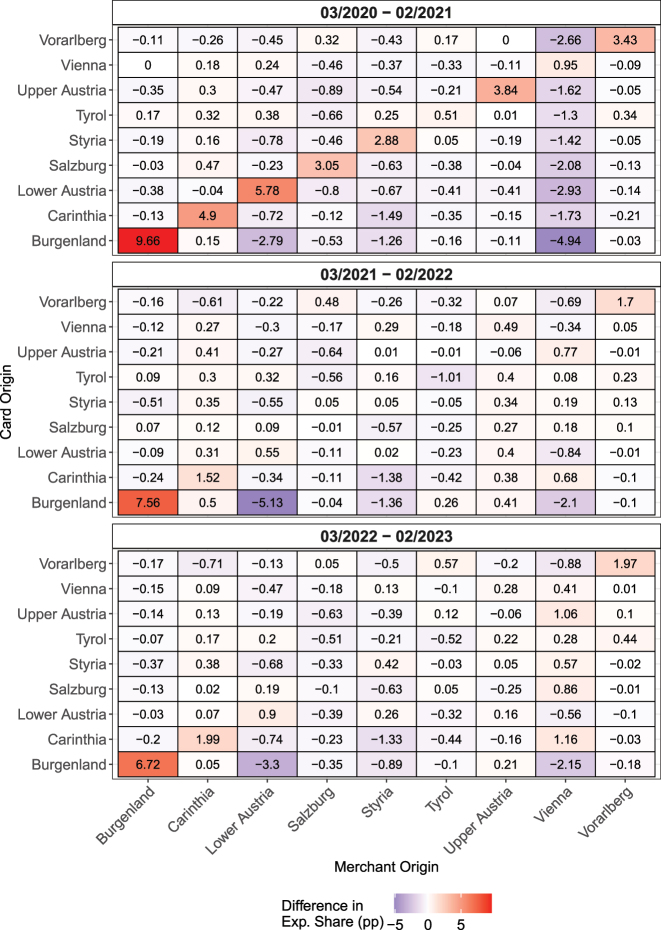
Changes of expenditures by merchant location and cardholder residence, during the COVID-19 pandemic and beyond (Austria). Notes: Changes of expenditure shares in percentage points, at location of merchants by residence of cardholders. The benchmark pre-pandemic period is 03/2019–02/2020. Source: Fable Data.

**Figure 21:**
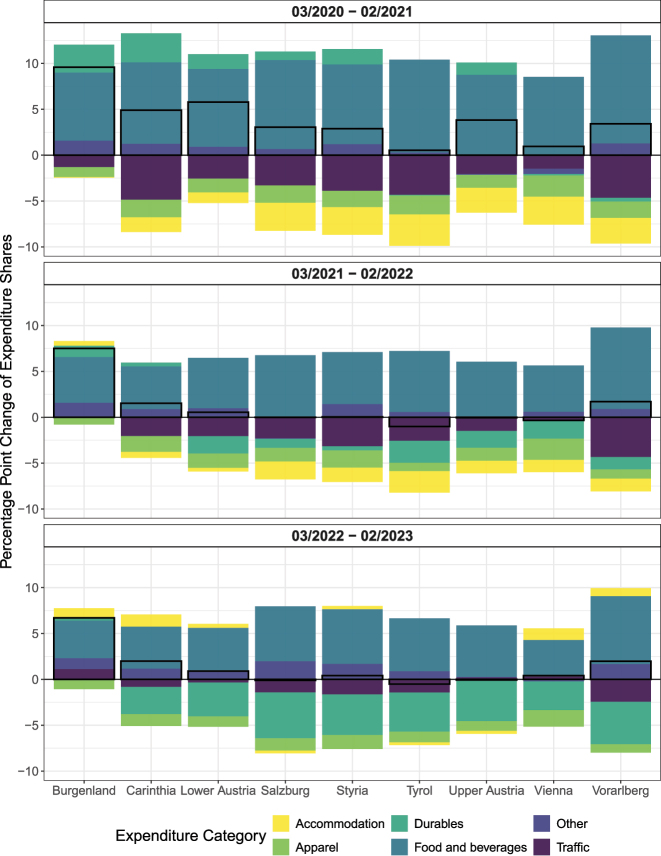
Decomposing the change of expenditure shares of cardholders in their region of residence (Austria). Notes: The black rectangles in the figure illustrate the total change of expenditures. Changes of expenditure shares of cardholders in percentage points, at merchants in the region of the residence of cardholders. The classification of durable expenditure is documented in Table 7, Appendix A.1. Source: Fable Data.

Figure 15 shows the weekday spending patters for durables (top panel), semi-durables (middle panel), and non-durables (bottom panel). Expenditures per weekday are displayed on the left for Germany and on the right for Austria, in a window of two weeks before and after Black Friday prior to the pandemic (2017–2019), during the pandemic (2020–2021), and after the pandemic (2022–2023). The figure delivers three main insights.

Firstly, the spending patterns are remarkably similar in Germany and Austria. Indeed, the correlation between weekday spending patterns for each expenditure category in the respective subperiod is very high across the two countries, with a correlation coefficient above 0.95 in seven out of nine category-subperiod combinations. The correlation has been a bit lower during the pandemic for durables and semi-durables, for which the correlation coefficient is 0.85 and 0.84.

Secondly, spending on Black Friday matters for spending on durables and semi-durables but does not change the weekday spending patterns of non-durables. At a daily frequency, there does not seem to be a strong complementarity or substitutability of spending for non-durables when discounts incentivize spending for durables or semi-durables around Black Friday.

Thirdly, spending for durables and semi-durables during the week of Black Friday have started to spread out towards earlier days in the same week in the later subperiods in the sample.25The expenditures displayed in the Figure 15 are so precisely estimated that the differences of expenditures between the subperiod 2017–2019 and the following subperiods are significant at conventional levels. The precision is such that 99 %-confidence intervals would not be visible in the figure. A similar pattern has been documented for Switzerland in Bruhin et al. (2025) and may reveal different pricing and purchasing strategies of businesses and consumers over time.

## 6 Conclusions

We have analyzed new transactional data for Austria and Germany provided by Fable Data. We have shown that the high frequency and regional granularity make these data a very useful complement to existing official statistics. Our analysis has provided new insights about shifts of expenditures within the consumption basket and across geographic locations in the recent crisis of the COVID-19 pandemic, and beyond. We have illustrated the potential of the data for understanding strategic decisions of businesses and households, by analyzing spending patterns at the daily frequency.

The increase of card usage over time in countries like Austria and Germany, as well as the planned extensions of the Fable data set to cover items in the consumption basket that are typically paid by bank transfers, such as rents for housing or car purchases, are likely to make this data source even more valuable in the future. As an important shortcoming remains the lack of information on the socio-demographic characteristics of card users.

## A Appendix

### A.1 Information on the Data

Table 5 lists the main variables we use in the Fable data set on the type of transactions and the type of users. Table 6 compares the age distribution in the sample and the population. Table 7 contains the mapping between the MCCs and the consumption categories of non-durables, semi-durables and durables.

**Table 5:** Description of main data attributes.

Transaction attributes	
**Attribute**	**Description**
TXN date	Date of transaction (TXN)
TXN key	Unique TXN key created by Fable to identify a TXN
Spend In	Refund or account credit
Spend Out	Card or account debit/spend
MCC	Merchant category code
**User attributes**	
User key	Unique user key created by Fable to identify a user
Age group	Age group of the user in 10-year age bins ( < 20, 20–29, …, 60–69, >=70 )
User postal sector	Postcode of user
User country	Country of user residence
**Merchant attributes**	
Merchant name	Name of the merchant
Merchant postcode	Postcode of merchant
Merchant country	Country of merchant

**Table 6:** Comparison of age distribution in the sample and the population.

Age	Germany		Austria	
	Sample	Population	Sample	Population
20–29	17.9	14.0	16.8	15.3
30–39	25.2	16.0	25.5	17.0
40–49	19.5	14.8	22.2	16.4
50–59	19.8	19.6	20.2	19.4
60–69	11.7	15.8	10.7	14.5
≥70	5.9	19.7	4.7	17.5

Notes: Shares of the population aged 20 and older. Source: Fable Data;population statistics available at Statistik Austria, https://www.statistik.at/statistiken/bevoelkerung-und-soziales/bevoelkerung/bevoelkerungsstand/bevoelkerung-nach-alter/geschlecht, and Destatis,. https://www-genesis.destatis.de/datenbank/online/table/12411-0005/table-toolbar, accessed in March 2025.

**Table 7:** Mapping of MCCs into the durable category.

Category		MCCs
Non-durable		5411, 5422, 5441, 5451, 5462, 5499
Semi-durable		5137, 5139, 5611, 5621, 5631, 5641, 5651, 5655, 5661, 5681, 5691, 5697, 5698, 5699, 5949, 7251, 7296
Durable	Electronics	5044, 5045, 5946, 5065, 5722, 5732, 5733, 5734, 7622, 7623, 7629
	Furniture	5021, 5712, 7641
	Home improvement	1520, 1711, 1731, 1740, 1750, 1761, 1771, 1799, 5039, 5072, 5074, 5198, 5200, 5211, 5231, 5251, 5261, 5713, 5714, 5718, 5719, 5996
	Automotive	5013, 5271, 5511, 5521, 5532, 5533, 5561, 5571, 5592, 5598, 5599, 7531, 7534, 7535, 7538
	Other durables	5094, 5099, 5932, 5940, 5941, 5942, 5944, 5945, 5948, 5950, 5971, 5972, 5998, 7631

Figure 16 shows that the expenditures shares in each region (as percent of the total expenditure in the respective country) are similar in the Fable Data and in the official statistics provided by the respective statistical offices in Austria and Germany. The figure plots the expenditures shares in the Fable Data for the sample period 2017–2023, for all expenditures in the Fable Data that have a counterpart in the consumption measure of the national accounts. The data from the statistical offices on the regional expenditures are available from the following sources for certain years:

– Germany (statistikportal.de, 2022): *Volkswirtschaftliche Gesamtrechnungen der Länder – Entstehung, Verteilung und Verwendung des Bruttoinlandsprodukts in den Ländern der Bundesrepublik Deutschland 1991 bis 2023, Reihe 1, Länderergebnisse Band 5, Reiter 7.19 (Private Konsumausgaben in jeweiligen Preisen je Einwohnerin bzw Einwohner)*
– Austria (Statistik Austria, 2019/2020): *Konsumerhebung 2019/2020, Tabelle – Monatliche Verbrauchsausgaben 2019/20 mit Schwankungsbreite – Bundesländer (Äquivalenzausgaben)*

### A.2 Expenditure Shares of Items Covered in the Fable Data Based on MCC

Figure 17 displays the expenditure shares in the Fable Data of those categories, for which we provide a time series comparison with official statistics based on revenues from firms in Section 3.2, which are available at monthly or quarterly frequency. The category *Other* is the residual category containing all other transactions.

### A.3 Card Transactions in Subsample Periods

Table 8 displays transaction amounts and cash withdrawals in the Fable Data over time.

**Table 8:** Transaction amounts and cash-withdrawals for cards in the Fable Data over time.

	Transaction amount		Cash withdrawals	
	Germany	Austria	Germany	Austria
2017–2019	67.77	55.91	177.62	140.67
2020–2021	58.27	52.42	203.99	152.65
2022–2023	59.50	54.82	198.27	155.89

Notes: Average amounts for active cards in the Fable Data during the respective sample periods. Source: Fable Data.

### A.4 Evidence for Austria

Figures 18– 21 provide results for Austria, which we have presented for Germany in the main text.

### A.5 Summary Statistics for Intensive User Sample

Table 9 compares summary statistics on age, income, and the location in the full sample and the intensive-user sample.

**Table 9:** Comparison of intensive-user sample with full sample, by country.

Variable	Germany		Austria	
	Intensive card-user sample	Full sample	Intensive card-user sample	Full sample
Age	46.03	45.09	46.54	44.64
Income (in 1,000€)^a^	34.85	34.69	29.31	29.22
Location (city share)	43.0 %	41.1 %	38.4 %	34.2 %

Notes: ^a^Average annual amount in the sample period 2017–2023. For income, the average is the population-weighted average of the mean income in each county. Location is a dummy that takes the value of 1 if the card user resides in a city (*Kreisfreie Stadt* (DE) or *Statutarstadt* (AT)), and takes the value of 0 otherwise. The full sample consists of active cards used at least once per year. The intensive-card user sample consists of cards used for at least 50 transactions and four different MCCs, with at least 1,500 euro spent on groceries per year (13 months for the construction of overlapping samples described in Section 4.4). Sources: Fable Data, Statistik Austria and Destatis (*Lohn- und Einkommensteuerstatistik*).

### A.6 Information on Additional Data Sources

#### A.6.1 National Accounts

In Figure 7, we compare expenditure baskets in transaction data to the average expenditure basket in *Ergebnisse der Laufenden Wirtschaftsrechnungen (LWR) – Haushaltsbuch*, Statistisches Bundesamt (Germany) and *Volkswirtschaftliche Gesamtrechnungen 1995–2022, Hauptergebnisse*, Statistik Austria (2022).

In Figure 8, we compare expenditure in Fable Data to the time series of final consumption expenditure of households (Eurostat, *namq-10-fcs*) in euro.

To compute the shares of the population aged 20 and older, we use the population statistics provided by Statistik Austria, and Destatis. For average income net of taxes at the municipality and NUTS3 level, we use the *Integrated wage and income tax statistics* from Statistik Austria and Destatis.

#### A.6.2 Revenue Statistics

We take the official indices in Section 3.2 from the national short-term business statistics. Indices on services are taken from the nominal monthly or quarterly turnover in services (Eurostat, *sts_setu_q*). We use *NACE55 – Accommodation* as the reference for accommodation, *NACE 56 – Gastronomy* for food services, and *NACE R2 – Information and communication* for communication. The indices for transportation are based on monthly air passenger transport (Eurostat, *avia_paoc*). Nominal wholesale and retail trade volume indices are available at the monthly or quarterly level (Eurostat, *sts_trtu_m*). We use *G47 – Retail sale of food, beverages, and tobacco* as the reference for groceries, *G47 – Retail sale of textiles, clothing, footware and leather goods in specialised stores* for clothing, and *G473 – Retail sale of automotive fuel in specialised stores* for fuel. For all revenue statistics, we use the time series at the highest frequency available.

Table 10 provides an overview for the data frequency of the external data used for the time series comparison.

**Table 10:** Frequency of external data source used for time series comparison.

Expenditure category	Germany	Austria
Groceries	Monthly	Monthly
Food & beverage services	Monthly	Quarterly
Clothing	Monthly	Monthly
Accommodation	Monthly	Quarterly
Communication	Monthly	Quarterly
Transportation	Monthly	Quarterly
Fuel	Monthly	Monthly

#### A.6.3 Payment Statistics

We use the following payment statistics as a comparison for Fable Data. We obtain the transaction (amounts) per card by dividing the aggregate transaction (volume) by the aggregate number of cards used.

**Cash withdrawals:**

– **Germany** (BuBa, 2022–2023)
  – **Debit Card**: *Zahlungstransaktionen nach Zahlungsinstrumenten/Bargeldabhebungen mit Karten/Bargeldabhebungen mit Debitkarten*
  – **Credit Card**: *Zahlungstransaktionen nach Zahlungsinstrumenten/Bargeldabhebungen mit Karten/Bargeldabhebungen mit Kreditkarten mit Kreditfunktion*
  – **Credit Card (broad)**: *Zahlungstransaktionen nach Zahlungsinstrumenten/Bargeldabhebungen mit Karten/Bargeldabhebungen mit Kreditkarten mit Kreditfunktion + Bargeldabhebungen mit Kreditkarten ohne Kreditfunktion*
– **Austria** (OeNB, 2017–2023)
  – **Debit Card**: *Geldausgabegeräte (ATM)/Behobene Beträge On-Us am Geldausgabeautomaten in Mio EUR/Anzahl der Behebungen On-Us am Geldausgabeautomaten in 1.000*
  – **Credit Card & Credit Card (broad)**: *Zahlungssystembetreiber – Delayed Debit- und Kreditkarten mit Bezahl- bzw. Bargeldfunktion/Beträge in Mio EUR Bargeldfunktion (behobene Beträge)/Anzahl der Transaktionen in Mio Bargeldfunktion (Behebungen)*

**Transaction Amount:**

– **Germany** (DB-nomics, 2017–2021)
  – **Debit Card** *Zahlungen mit Debitkarten*
  *BBBZ1.A.ZV00.INET.I10.T10.I21.TO.A1.V/N*
  – **Credit Card** *Zahlungen mit Kreditkarten (mit Kreditfunktion)*
  *BBBZ1.A.ZV00.INET.I10.T10.I23.TO.A1.V/N*
  – **Credit Card (broad)** *Zahlungen mit Kreditkarten (mit Kreditfunktion) + Zahlungen mit Kreditkarten (ohne Kreditfunktion)*
  *BBBZ1.A.ZV00.INET.I10.T10.I23.TO.A1.V/N*
  *+ BBBZ1.A.ZV00.INET.I10.T10.I22.TO.A1.V/N*
– **Austria** (OeNB, *Zahlungskartentransaktionen mit in Österreich ausgegebenen Karten*, 2017–2023)
  – **Debit Card**: *Beträge/Anzahl der Zahlungstransaktionen von Österreichern in Mio EUR mit Debitkarte in Österreich + im Ausland*
  – **Credit Card**: *Beträge/Anzahl der Zahlungstransaktionen von Österreichern in Mio EUR mit Kreditkarte mit Kreditfunktion in Österreich + im Ausland*
  – **Credit Card (broad)**: *Beträge/Anzahl der Zahlungstransaktionen von Österreichern in Mio EUR mit Kreditkarte ohne Kreditfunktion (Delayed Debitkarte)/mit Kreditfunktion in Österreich + im Ausland*

**Cards per Household:** In 2023, households had on average:

– **Germany:** 4.28 cards (3.44 debit cards, 0.84 credit + delayed debit cards),
– **Austria:** 3.50 cards (2.68 debit cards, 0.82 credit + delayed debit cards).

These statistics are obtained using the following data series:

– **Number of Households:** eurostat – lfst_hhnhtych
– **Germany** (ECB Dataportal, *Number of Payment Cards*, 2023)
  – **Total Number:** *[PCN.A.DE.PCS_ALL.1.PN] Number of payment cards in circulation (except cards with e-money function only) – issued by resident PSPs in: Germany, Germany, Annual*
  – **Debit Cards:** *[PCN.A.DE.PCS_ALL.11.PN] Number of debit cards – issued by resident PSPs in: Germany, Germany, Annual*
  – **Delayed Debit Cards:** *[PCN.A.DE.PCS_ALL.12.PN] Number of delayed debit cards – issued by resident PSPs in: Germany, Germany, Annual*
  – **Credit Cards:** *[PCN.A.DE.PCS_ALL.13.PN] Number of credit cards – issued by resident PSPs in: Germany, Germany, Annual*
– **Austria** (DB-nomics, *Number of Payment Cards*, 2023)
  – **Total Number:** *[A.AT.PCS_ALL.1.PN] Annual – Austria – ALL payment card schemes – Payment function (except e-money function only) – Pure number (PN)*
  – **Debit Cards:** *[A.AT.PCS_ALL.11.PN] Annual – Austria – ALL payment card schemes – Debit card – Pure number (PN)*
  – ***Delayed Debit Cards:*** *[A.AT.PCS_ALL.12.PN] Annual – Austria – ALL payment card schemes – Delayed debit card – Pure number (PN)*
  – **Credit Cards:** *[A.AT.PCS_ALL.13.PN] Annual – Austria – ALL payment card schemes – Credit card – Pure number (PN)*
